# Pharmacological Neuroenhancement: teachers’ knowledge and attitudes–Results from a survey study among teachers in Germany

**DOI:** 10.1186/s13011-016-0077-y

**Published:** 2016-09-20

**Authors:** Andreas G. Franke, Sophie Lehmberg, Michael Soyka

**Affiliations:** 1 Department of Social Work and Education, University of Neubrandenburg (University of Applied Sciences), Brodaer Str. 2, 17033 Neubrandenburg, Germany; 2 Private Clinic Meiringen, Clinic for Psychiatry and Psychotherapy, 3860 Meiringen, Switzerland; 3 Ludwig Maximilian University (LMU), Nussbaumstr. 7, 80336 Munich, Germany

**Keywords:** Neuroenhancement, School, Teachers, Public health concern, Misuse

## Abstract

**Background:**

Pharmacological neuroenhancement (PN) is a topic of increasing importance. Its prevalence rates range from 1 % to more than 20 %. Students are a group that shows exceptionally high prevalence rates. However, little is known about teachers’ knowledge, management, attitudes and ethical judgements regarding PN.

**Methods:**

A web-based survey containing 40 closed questions was developed. All teachers working at all private and public schools in Mecklenburg-Vorpommern, a state in northeastern Germany, were invited to participate after their respective school offices were contacted by telephone, email and mail.

**Results:**

In total, 255 teachers participated in the survey. Of those, 73.3 % had already heard about PN in general, and 68.2 % had heard about PN in students. Their sources of knowledge were digital media such as TV (73.8 %) and the internet (40.6 %) and print media (64.7 %); their own students informed 29.9 % of the teachers about PN in general and 35.6 % of them about PN among students. Furthermore, 34.9 % of the surveyed teachers were convinced that PN substance use was ineffective in general, and 51.8 % of the surveyed teachers believed that PN substances were ineffective in achieving better grades. Only 1.2 % thought that none of the so-called PN substances could lead to addiction, and 37.6 % would classify PN substance use as general drug misuse. The highest values regarding risk of addiction were observed for illicit drugs. The prevalence of PN substance use was evaluated to be very low and to be significantly higher in male, highly skilled and college/university students. In total, 1.6 school lessons per year were used to discuss PN. Finally, 55.7 % of the surveyed teachers believed that performance-enhancing substances should be forbidden at schools.

**Conclusion:**

Teachers, as an integral part of the education of children and adolescents, often know about PN substances and mostly refuse their use being afraid about the risk of addiction. However, regarding effects as well as side effects of PN substances, teachers have very different opinions. Furthermore, they seem to underestimate the prevalence among their students and broach the topic infrequently. Teachers should be sensitized for high prevalence rates and should broach the topic of PN more frequently to their students to prevent potential misuse of PN substances.

## Background

The number of scientific publications and public press releases in magazines and newspapers on pharmacological neuroenhancement (PN) have been substantially increasing each year for more than the past 10 years [1]. One of the first publications about the misuse of stimulants assessing i.a. prevalence rates of PN was conducted by Timothy Wilens and colleagues, who demonstrated a past-year prevalence rate of 5–35 % for the general misuse of “stimulants prescribed for ADHD” (attention deficit hyperactivity disorder) such as amphetamines (AMPH) and methylphenidate (MPH) among college students in a systematic review [2]; however, PN was only one of the reasons listed for stimulant use. Additionally, numerous studies from different countries have examined PN among students and shown prevalence rates of PN of approximately 1 to 20 %, depending on the substances assessed and the survey methods used [3–9]. One of the most recent epidemiological studies found a prevalence rate of 13 % for “the use of prescription drugs, alcohol, or illegal drugs for CE” (cognitive enhancement) among secondary school students [10]; however, they assessed the use of several potential PN substances such as “prescription drugs, recreational drugs, and ‘Soft Enhancers’”. Anonymous epidemiological studies among adults (e.g. scientists, surgeons) have shown lifetime prevalence rates of up to 20 % for prescription and illicit drugs and often even higher rates for caffeine [11–14].

Regarding the correlation between PN substance use and a student’s environment, studies have shown that academic background affects PN substance use: students used PN substances (mainly stimulant drugs) during high-stress periods such as preparation periods for exams, to stay awake longer or to study more effectively with the aim of better performance and better grades [4, 6, 15]. A waste water analysis of a student dormitory confirmed these assumptions; Burgard and colleagues found high rates of stimulant medication (AMPH, MPH) metabolites during preparation periods prior to exams [16]. Furthermore, there seems to be a correlation between substance misuse or addiction in general and substance use for PN [17–19]. However, student users state being aware of the risks of addiction in using PN substances [15, 20, 21]. Nevertheless, the putative aspects of misuse and addiction regarding PN lead to the assumption that PN could be or at least become a public health concern.

Different aspects of the attitudes and ethics regarding PN have been studied among student and non-student samples, as recently reviewed by Schelle and colleagues [22].

An anonymous survey of 1400 university teachers in Germany revealed that this group had a low willingness to use PN substances themselves, which was caused mainly by their fear of side effects and furthermore by their internalized norms [23]; the study is based on the “willingness to take various hypothetical CE-drugs” and researchers varied the description of the CE drug towards their participants. Additionally, the same authors demonstrated an association between the perceptions of work-related stress and PN substance use among university teachers [24]. Moreover, a recent survey study by Wolff and colleagues of Jordanian employees, a majority of whom were teachers (723 teachers among all 1186 surveyed employees), revealed a 12-month prevalence rate of 15.4 % for the use of prescription drugs for PN using an anonymizing technique; the subsample of teachers showed an even higher prevalence rate of 26.2 % [25]. Outside these two studies, at least to our knowledge, there have been no published data about PN in teachers, who are – given the scope of their roles in schools – instrumental in conveying information, attitudes and norms about different topics to their pupils/students.

In the scientific community dealing with PN, there is no homogenous definition of PN. For the purpose of this study PN is described to the participants as any kind of substances used by healthy people without medical need and with the aim of increasing their cognitive skills.

The present web-based study was designed to collect primary data on teachers’ knowledge, management, attitudes and ethical judgements regarding PN among pupils/students, who seem, according to international data, to be the largest group of PN substance users.

Being a web-based survey the study enables a high degree of anonymity and “honest” answers can be expected, but the study design limits its explanatory power as participation could not have been controlled (participation/ response bias); therefore no hypothesis can be given for the absolute level of knowledge and attitudes.

## Methods

The present study was designed as an online survey using the survey tool “Unipark”. School offices of the following schools were contacted:

- all public and private elementary schools (*n* = 46) (German term: “Grundschule”; from class 1 – 4, students being 6 – 10 years old),
- all public (*n* = 59) and private (*n* = 5) grammar schools (German term: “Gymnasium”; class 5 – 12, students being 10 – 19 years, education preparing for college/ university)
- all vocational schools (*n* = 56) (German term: “Berufsschule”; class 9 – 12, students being 16 – 19 years, preparing for vocational training/ apprenticeship) as well as
- all comprehensive schools with secondary school (*n* = 43 (German term: “Gesamtschule”; grade 5 – 12, students being 10 – 19 years, mixture of classes preparing for college/ university and classes preparing for succeeding vocational education).

All were contacted via 1) telephone, 2) email and 3) mail and were informed about the survey and instructed to distribute information about the survey to all respective teachers. The invitation process took place in September 2014, and the survey was conducted between September and November 2014.

### Data acquisition

To ensure a high degree of privacy and anonymity, the survey was designed as an online poll. The questionnaire began with eight independent questions/ independent variables concerning participants’ characteristics: sex (male, female), age (clustered: 20 – 29 years, 30 – 39 years, 40. – 49 years, 50 – 59 years, 60 years and older), having own children (dichotomous), age of own children, working at which type of above-mentioned school (private or public, elementary comprehensive, vocational, grammar school), school subject/s taught, years working as a teacher) and 32 questions about the study subject as follows. Prior to the questions PN was explained to be the use of substances of any kind by healthy people without medical need with the aim of increasing cognitive skills e.g. vigilance, concentration, memory, etc.

The questions addressed participants’

- knowledge about PN: having ever heard about PN, sources of knowledge (print media, TV, internet, colleagues, friends/ relatives, students, during college time, free space to add another source), substances belonging to the group of PN substances (list of OTC (over the counter) substances (e.g. Ginkgo biloba, caffeine tablets), prescription drugs (e.g. Ritalin®, Adderal®) and illicit drugs (e.g. “Speed”, Exstasy) and free space to add substances), risk of getting addicted (in general and for each substance by the list mentioned before), effects and side effects of PN substances (in general and for each substance by the list mentioned before),
- knowledge about use of PN substances among pupils/students, anticipated frequency of use among students of the different PN substances (in general and for each substance by the list mentioned before), characteristics of students anticipated to use PN substances (age of use, context of use),
- consideration of PN in school lessons (frequency of dealing with PN during lessons, school subject dealing with PN substance use, age of students broaching PN) and
- attitudes as well as ethical aspects/judgements regarding PN (relevance of PN at school, type of focus (biological, ethical, etc.), il/legality of PN substance use, using PN substances in highly responsible professions such as pilots and physicians).

Multiple answers were allowed for many of the questions (*n* = 13).

### Data analysis

Data were collected and stored in the Unipark database. Statistical analyses were performed with SPSS for Windows, version 23.0. Binary regression analysis with stepwise forward selection was used to predict the dependent variables of the questionnaire. Continuous variables (e.g. age) were dichotomized. The results are presented as the means and standard deviations (SD), prevalence rates (%) or odds ratios (OR) with Clopper-Pearson confidence intervals (95 % CI) and p-values and degrees of freedom (df).

According to the distribution of the variables and univariate analyses, post-hoc tests were performed (Tukey procedure) or non-parametric tests (Kruskal-Wallis, *U*-test, Chi-Square-test) were used to test for significant differences. By fulfilling the statistical qualifications (normal distribution, homoscedasticity) and in case of continuous variables univariate analyses (ANOVA, t-tests) were used. If statistical qualifications were not fulfilled or in case of ordinal dependent variables, Kruskal-Wallis or U-tests were used. In case of nominal dependent variables Chi-Square tests were used to test for significant differences.

The percentages are based on the number of participants who answered the respective questions which is presented in the text as ZZ% (*n* = XX of *n* = YY).

## Results

In total, all public and private grammar and vocational schools as well as comprehensive schools with secondary school in Mecklenburg-Vorpommern were informed about the survey, and 255 teachers participated. 169 participants completed the full questionnaire and answered to all questions. Participants were mainly female (*n* = 163 of *n* = 230) and had an average age of 50 – 59 years (*n* = 110 of *n* = 234). A third (34.9 %, *n* = 89) had children living in the same household, who were an average age of 6.9 years old. A third of the participants had been employed for 20 – 29 years (29 %, *n* = 74 of *n* = 255). For further participant characteristics, see Table 1.

**Table 1:** Basic characteristics of the survey participants

Sex	Male: 26.3 % (*n* = 67)
	Female: 63.9 % (*n* = 163)
	Not specified: 9.8 % (*n* = 25)
Age	20–29 years: 11.4 % (*n* = 29)
	30–39 years: 12.2 % (*n* = 31)
	40–49 years: 24.3 % (*n* = 62)
	50–59 years: 43.1 % (*n* = 110)
	60 years and more: 0.8 % (*n* = 2)
	Not specified: 8.2 % (*n* = 21)
Children living in the same household	Yes: 34.9 % (*n* = 89)
	No: 53.3 % (*n* = 136)
	Not specified: 11.7 % (*n* = 30)
Schools	Public and private elementary schools: *n* = 46
	Public and private grammar schools: *n* = 64
	Vocational schools: *n* = 56
	Comprehensive schools with secondary school: *n* = 43
	Not specified: *n* = 46
Years worked as a teacher	1–9 years: 17.6 % (*n* = 45)
	10–19 years: 11.4 % (*n* = 29)
	20–29 years: 29 % (*n* = 74)
	30–39 years: 25.9 % (*n* = 66)
	40 years and more: 0.4 % (*n* = 1)
	Not specified: 15.7 % (*n* = 40)

### Knowledge of PN substance use

The majority (93.5 %, *n* = 187 of *n* = 200) who answered the question on their knowledge of PN substance use in the general population stated that they had already heard about PN in general; only 6.5 % (*n* = 13 of *n* = 200) responded that they had not heard about PN before. When asked about the source of their information, three-fourths of the responding participants had heard about PN on TV (73.8 %, *n* = 138 of *n* = 187) followed by from print media (64.7 %, *n* = 121 of *n* = 187). In addition, 40.6 % (*n* = 76 of *n* = 187) had been informed on the internet, 26.2 % (*n* = 49 of *n* = 187) by colleagues and 32.1 % (*n* = 60 of *n* = 187) by friends and relatives. A total of 29.9 % (*n* = 56 of *n* = 187) had heard about PN from their own students, and only 11.8 % (*n* = 22 of *n* = 187) knew about PN from their own time at university. Regarding the aspect knowledge of PN substance use, there were no significant differences in participants’ characteristics (sex, age, having own children, age of own children, working at which type of above-mentioned school, school subject/s taught, and years working as a teacher).

When asked about the use of PN substances among students, the majority of the surveyed teachers again had already heard about it (88.8 %, *n* = 174 of *n* = 196); only 11.3 % (*n* = 22 of *n* = 196) had not. The sources of this knowledge were TV (67.8 %, *n* = 118 of *n* = 196), print media (52.9 %, *n* = 92 of *n* = 196), the internet (32.8 %, *n* = 57 of *n* = 196), friends and relatives (30.5 %, *n* = 53 of *n* = 196), colleagues (25.9 %, *n* = 45 of *n* = 196) and their own regularly taught students (35.6 %, *n* = 69 of *n* = 196). Significantly more teachers with children living in their own household had heard about PN substances from their students than teachers who had no children (ANOVA, *p* = .004, CI: 05 -.32). Furthermore, significantly fewer 30- to 39-year-old teachers had heard about PN substances than had 20- to 29-year-old (ANOVA, *p* = .040, Cl: .01- .53) and 40- to 49-year-old teachers (ANOVA, *p* = .021, Cl: .02-.42), Table 2 shows data about the respective substances.

**Table 2:** Knowledge of teachers about substances used for PN in general and among students

PN substance	PN substance use in general (*n* = 197)	PN substance use among students (*n* = 169)
Ginkgo biloba	15.2 %	7.1 %
Energy drinks	91.9 %	87.6 %
Caffeine tablets	68 %	46.7 %
MPH	51.8 %	43.2 %
AMPH	25.9 %	13.6 %
Atomoxetin	4.6 %	4.1 %
Modafinil	2.5 %	1.8 %
Antidementia drugs	10.2 %	3 %
Illicit AMPH	48.7 %	27.8 %
Ecstasy/ MDMA	54.3 %	32.5 %
Cocaine	39.6 %	21.3 %
Ephedrin	17.8 %	9.5 %
Cannabis	47.7 %	32 %

77.3 % (*n* = 197 of *n* = 255) of the surveyed teachers answered “in general”; 66.3 % (*n* = 169 of *n* = 255) answered “among students”

*AMPH* amphetamines, *MPH* Methylphenidate, *PN* pharmacological neuroenhancement

Regarding the substances used for PN in general, most of the respondents had heard that energy drinks (91.9 %, *n* = 181 of *n* = 197) and caffeine tablets (68 %, *n* = 134 of *n* = 197) were used for PN. Furthermore, 54.3 % (*n* = 107 of *n* = 197) knew about illicit ecstasy/MDMA being used for PN, followed by 51.8 % about MPH (*n* = 102 of *n* = 197). There were no significant differences in the additionally surveyed independent variables. For further results, see Table 2.

### Knowledge about the effectiveness of PN substances

Regarding knowledge of the pro-cognitive effectiveness of PN substances use in increasing academic performance in general, 34.9 % (*n* = 59 of *n* = 169) of the respondents were convinced that none of the so-called PN substances had pro-cognitive effects. As far as specific substances, 42 % (*n* = 71 of *n* = 169) of the answering teachers were convinced that caffeine tablets had a certain effect, followed by 29.6 % regarding energy drinks (*n* = 50 of *n* = 169) and 25.4 % (*n* = 43 of *n* = 169) regarding MPH. Significantly more teachers with children living in their household than teachers without children were convinced that MPH had an effect (ANOVA, *p* = .011, Cl: .03 - .30). Further significant differences could not be detected. Detailed data of the respective substances is shown in Table 3.

**Table 3:** Pro-cognitive effectiveness of potential PN substances

PN substance	Pro-cognitive effectiveness for PN in general
No substance	34.9 % (*n* = 59)
Ginkgo biloba	7.1 % (*n* = 12)
Energy drinks	29.6 % (*n* = 50)
Caffeine tablets	42 % (*n* = 71)
MPH	25.4 % (*n* = 43)
AMPH	7.1 % (*n* = 12)
Atomoxetin	1.2 % (*n* = 2)
Modafinil	0 % (*n* = 0)
Antidementia drugs	1.2 % (*n* = 2)
Illicit AMPH	11.2 % (*n* = 19)
Ecstasy/ MDMA	11.8 % (*n* = 20)
Cocaine	11.2 % (*n* = 19)
Ephedrin	4.7 % (*n* = 8)
Cannabis	3 % (*n* = 5)

66.3 % (*n* = 169) answered that question

*AMPH* amphetamines, *MPH* Methylphenidate, *PN* pharmacological neuroenhancement

Regarding the possibility of achieving better grades (see Table 4) by using PN substances, 27.1 % (*n* = 69 of *n* = 255) of the surveyed teachers thought that it would be possible to achieve better grades by using PN substances in general. The majority of the surveyed teachers thought that it would be possible to achieve better grades through the use of prescription drugs. Half of the respondents were convinced that there was no possibility of achieving better grades through the use of PN substances. For further results see Table 4.

**Table 4:** Achieving better grades by the use of PN substances

Better grades by the use of PN substances	Yes	No
Over the counter substances	33.3 % (*n* = 23)	81.8 % (*n* = 108)
Prescription drugs	94.2 % (*n* = 65)	56.1 % (*n* = 74)
Illicit drugs	13 % (*n* = 9)	90 % (*n* = 120)

27.1 % (*n* = 69) answered that question with yes. 51.8 % (*n* = 132) answered that question with no

*PN* pharmacological neuroenhancement

Non-parametric tests revealed that the type of school in which teachers worked was significantly associated with the assumption that OTC substances would not lead to better school grades (Chi-Square test, *p* = .038, df = 4). Furthermore, teacher’s age was associated with this assumption (Chi-Square test, *p* = .047, df = 3). Additionally, school type was significantly associated with the assumption that prescription drugs were associated with better school grades (Chi-Square test, *p* = .026, df = 4).

### Assumptions about PN substance use leading to addiction

All participants were surveyed about their opinion of whether PN substances use could lead to addiction. Only 1.2 % (*n* = 2 of *n* = 168) of the respondents thought that none of the so-called PN substances could lead to addiction. Between one third and half of the participants thought that prescription drugs could lead to addiction (see Table 5); nearly all of the participants thought that the use of illicit drugs could lead to addiction, and less than one third thought that the aforementioned OTC substances as well as caffeinated/ energy drinks (e.g. Red Bull®) could lead to addiction. Further information is provided in Table 5. Regarding the independent variables, there were no significant differences.

**Table 5:** PN substances use leading to addiction

No substance	1.2 % (*n* = 2)
Ginkgo biloba	4.8 % (*n* = 8)
Energy drinks	19.6 % (*n* = 33)
Caffeine tablets	33.3 % (*n* = 56)
MPH	51.8 % (*n* = 87)
AMPH	42.9 % (*n* = 72)
Atomoxetin	32.1 % (*n* = 54)
Modafinil	30.4 % (*n* = 51)
Antidementia drugs	33.3 % (*n* = 56)
Illicit AMPH	86.9 % (*n* = 146)
Ecstasy/ MDMA	88.7 % (*n* = 149)
Cocaine	95.8 % (*n* = 161)
Ephedrin	74.4 % (*n* = 125)
Cannabis	82.7 % (*n* = 139)

65.9 % (*n* = 168) answered that question

*AMPH* amphetamines, *MPH* Methylphenidate, *PN* pharmacological neuroenhancement

### Anticipated prevalence rate of PN substance use

All participants were asked about their appraisal of the prevalence of PN substances among students over 18 years (very widespread, fairly widespread, scarcely widespread) and about the characteristics of the students using PN substances. Overall, teachers rated the prevalence of PN substances to be very low, with Ginkgo biloba being the most widespread substances for PN, and no one thought illicit AMPH to be widespread or fairly widespread. Further results about the anticipated distribution of PN substances are presented in Table 6.

**Table 6:** Anticipated distribution of PN substances

PN substance	Very wide spread	Fairly spread	Scarcely spread
Ginkgo biloba	9 % (*n* = 23)	16.1 % (*n* = 41)	8.2 % (*n* = 21)
Energy drinks	0.8 % (*n* = 2)	5.9 % (*n* = 15)	25.5 % (*n* = 65)
Caffeine tablets	2 % (*n* = 5)	12.9 % (*n* = 33)	18 % (*n* = 46)
MPH	2 % (*n* = 5)	7.5 % (*n* = 19)	6.7 % (*n* = 17)
AMPH	0.4 % (*n* = 1)	2.7 % (*n* = 7)	12.5 % (*n* = 32)
Atomoxetin	1.2 % (*n* = 3)	7.1 % (*n* = 18)	7.1 % (*n* = 18)
Modafinil	0 % (*n* = 0)	2 % (*n* = 5)	12.9 % (*n* = 33)
Antidementia drugs	0 % (*n* = 0)	6.7 % (*n* = 17)	8.6 % (*n* = 22)
Illicit AMPH	0 % (*n* = 0)	0 % (*n* = 0)	13.3 % (*n* = 34)

65.5 % (*n* = 167) answered that question

*AMPH* amphetamines, *MPH* Methylphenidate, *PN* pharmacological neuroenhancement

Non-parametric tests (Kruskal Wallis test) showed that the time participants had been a teacher was associated with the assumption that the use of Ginkgo biloba among students was widely spread (*p* = .047, df = 3) and that AMPH use was scarcely spread (*p* = .040, df = 3).

Regarding the characteristics of students using PN substances as dichotomous variables, teachers stated that they thought that significantly more highly skilled students (19.6 %, *n* = 50 of *n* = 255) would use PN substances than students with lower academic skills (9.4 %, *n* = 24 of *n* = 255) (*t*-test, *p* < .001, Cl: 2.23–2.61; df = 181). Teachers thought that significantly more male (36.9 %, *n* = 94 of *n* = 255) than female students (9 %, *n* = 56 of *n* = 255), (*t*-test, *p* < .001, Cl: 1.56– 1.84; df = 180) used PN substances as well as a significantly higher proportion of college and university students (48.6 %, *n* = 124 of *n* = 255) than school students/ pupils (4.3 %, *n* = 11; of *n* = 255, *t*-test, *p* < .001, Cl.: 1.96–2.15, df = 179).

Significantly more teachers in vocational schools than teachers of other schools thought that their students used PN substances in general (Kruskal-Wallis test, *p* = .046, df = 4). Significantly more teachers in public grammar schools than other teachers thought that students who used (psychoactive) drugs in their leisure time also used PN substances (Kruskal-Wallis test, *p* = .014, df = 4).

Furthermore, we asked all participants about their estimates of the frequency (“once”, “occasionally” and “regular”) of PN substance use among high school students not less than 18 years old and posed three questions: the first about the anticipated frequency for OTC substances, the second for prescription drugs and the third for illicit drugs. We offered free space for the participants to write percentages down as to how many students they would think would use the respective substance group “once”, “occasionally” and “regular”. For these percentages we present the respective percentage as mean and standard deviations: 29.4 % (*n* = 75 of *n* = 255) of the surveyed teachers noted their figures down and estimated that 43.4 % +/-30.27 of the above mentioned students would have already used an OTC substance or an energy drink “once”; asked for prescription drugs (28.2 %, *n* = 72 of *n* = 255 answered to that question), the answering teachers thought that 9.0 % +/- 8.64 of the above mentioned students would have used a prescription drugs “once”. Regarding illicit drugs, the answering teachers (28.2 %, *n* = 72 of *n* = 255) estimated that 18.3 % +/- 17.1 of high school students of 18 years and older had already used illicit drugs once.

On the second question about the anticipated occasional substance use, the surveyed teachers (27.4 %, *n* = 70 of *n* = 255 answered to that question) stated (by writing down percentages) that 36.8 % +/- 25.6 of the students would use OTC substances or an energy drink “occasionally”. 26.6 % (*n* = 68 of *n* = 255) of the surveyed teachers answered to the question of occasional use of prescription drugs and estimated that 7.2 % +/- 7.8 of the students would use prescription drugs occasionally. For illicit drugs (27.8 %, *n* = 71 of *n* = 255 answered) teachers estimated that 13.8 % +/- 14.0 of the students (high school students of 18 and older) would use illicit drugs occasionally.

Finally, for “regular” substance use for PN, teachers thought 24.7 % +/- 24.1 of the respective students to use OTC substances and energy drinks “regularly” (27 %, *n* = 69 of *n* = 255 teachers answered to that question). 4.2 % +/- 5.8 estimated a regular prescription drug use for PN among the respective students (25.9 %, *n* = 66 of *n* = 255 teachers answered). Furthermore, the surveyed teachers (29 %, *n* = 74 of *n* = 255 answering to the respective question) estimated that 7.5 % +/- 9.6 of the high school students not less than 18 years old used illicit drugs regularly.

### Interactions with students and addressing PN at school

Of all the participants, 50.2 % (*n* = 128 of *n* = 255) did not talk about PN during their lessons, and only 14.9 % (*n* = 38 of *n* = 255) reported broaching the subject of PN in their school lessons. In a slightly higher proportion talked about PN in grades 5 – 9 (31.6 %, *n* = 12 of *n* = 38); and a significantly higher number of teachers talked about PN in grades 10 – 13 (89.5 %, *n* = 34 of *n* = 38). When asked about the school subject in which they had discussed PN, social sciences, philosophy and biology were the subjects in which PN was talked about the most. As for the number of lessons that addressed PN, the mean value was 1.60 +/- 1.74 school lessons. Regarding the emphasis of the lessons on PN, effects, side effects and the risk of addiction were the main emphases. For further results, see Table 7. There were no significant differences in the independent variables.

**Table 7:** Emphasis of lessons when talking about PN

Effects	81.6 % (*n* = 31)
Side effects	76.3 % (*n* = 29)
Risk of addiction	81.6 % (*n* = 31)
Warning of intake	68.4 % (*n* = 26)
Epidemiology	2,6 % (*n* = 1)
Information about PN	63.2 % (*n* = 24)
Information source	10.5 % (*n* = 4)
Helpline	47.4 % (*n* = 18)

14.9 % (*n* = 38) answered that question

*PN* pharmacological neuroenhancement

In total, 41.6 % (*n* = 106 of *n* = 255) thought that PN was an important topic at their schools, and 11 % (*n* = 28 of *n* = 255) did not. The rest (*n* = 121 of *n* = 255) did not answer that question. There were no significant differences in the independent variables. Finally, 39.2 % (*n* = 100 of *n* = 255) of all surveyed teachers reported being interested in further training for themselves about PN, and 13.3 % (*n* = 34 of *n* = 255) were not interested.

### Attitudes towards PN

Teachers were also asked about the “status” of PN substances in schools. Half of the surveyed teachers (55.7 %; *n* = 142 of *n* = 255) believed that performance-enhancing substances in general should be prohibited at schools. Additionally, 59.2 % (*n* = 84 of *n* = 142) of the answering teachers thought that the use of OTC substances and energy drinks should be forbidden, 56.3 % (*n* = 80 of *n* = 142) felt the use of prescription drugs should be forbidden, and 99.3 % (*n* = 141 of *n* = 142) thought that the use of illicit drugs should be forbidden. Significantly more teachers who worked in public grammar schools believed that one should forbid the use of prescription drugs than did teachers at grammar schools (ANOVA, *p* < .001, Cl: .22 - .77). Significantly more teachers who worked in comprehensive schools with a secondary school supported forbidding the use of prescription drugs than did grammar school teachers (ANOVA, *p* < .001, Cl: .18 - .077), and significantly more vocational school teachers than grammar school teachers supported the opinion that prescription drugs should be forbidden (ANOVA, *p* = .019, Cl: .03 - .59). Regarding the question of forbidding substances for physical enhancement in sports clubs and competitions (the term “doping” was not used in this question), 70.7 % (*n* = 111 of *n* = 158) of the answering teachers stated that in this context, OTC substances and energy drinks should be forbidden, 85.4 % (*n* = 135 of *n* = 158) believed that prescription drugs should be forbidden and 99.4 % (*n* = 157 of *n* = 158) said illicit drugs should be forbidden in the aforementioned context. Significantly more of the older teachers (50 – 59 years) stated that they would forbid OTC substances and energy drinks for physical enhancement than did younger teachers (aged 20 – 29 years) (Kruskal-Wallis test, *p* = .022, df = 3).

Teachers were also asked how they would classify PN substance use; 37.6 % (*n* = 96 of *n* = 255) would classify PN substance use as general (psychoactive) drug misuse, 23.9 % (*n* = 61 of *n* = 255) classified PN substance use as independent from (psychoactive) drug misuse in general, and 4.7 % (*n* = 12 of *n* = 255) could not decide. The rest (*n* = 86 of *n* = 255) did not answer that question. There were no significant differences in the independent variables.

When comparing the use of caffeine tablets to the use of prescription drugs for PN, 32.9 % (*n* = 84 of *n* = 255) of the teachers stated that OTC substance and energy drink use was “something other” than prescription drug use for PN, 25.1 % (*n* = 64 of *n* = 255) rated OTC substance and energy drink use and prescription drug use for PN as equal and 8.3 % (*n* = 21 of *n* = 255) could not decide. The rest (*n* = 86 of *n* = 255) did not answer that question.

When asked about the use of PN substances in certain responsible professions, only 1.2 % (*n* = 3 of *n* = 255) and 0.4 % (*n* = 1 of *n* = 255) favoured the use of such drugs in pilots and physicians; 56.9 % (*n* = 145 of *n* = 255) and 58 % (*n* = 148 of *n* = 255) did not favour the use of PN substances in pilots and physicians, respectively. Again, there were no significant differences for the independent variables.

## Discussion

This study used an anonymous web-based questionnaire to investigate for the first time the knowledge, attitudes, ethical aspects and management of PN among teachers. We could demonstrate that the majority of the surveyed teachers had already heard about PN in general and about PN among students. Their sources of information were mainly print and digital media; however, one third was informed about PN even by their own students. Regarding pro-cognitive and other effects, there was disagreement: a third of the answering teachers thought that PN substance use was ineffective in general, and half of the surveyed teachers stated PN substance use was ineffective in achieving better grades. The vast majority thought that PN could lead to addiction and a third would classify PN substance use in the context of general (psychoactive) drug misuse and half of the participants stated that PN substance use should be forbidden at schools. The highest values regarding the belief that a substance could lead to addiction was observed for illicit drugs. The prevalence of PN substance use was evaluated to be very low and to be significantly higher in male, highly skilled and college/university students. Only very few school lessons per year were used to discuss PN.

Defining in their paper a “CE-drug” as “pharmaceuticals, especially prescription drugs” and using Likert scales, Wiegel and colleagues found a very low willingness of university teachers to use PN substances defined above on their own [24]. Furthermore, the researchers demonstrated that PN substance use among these participants was strongly associated with the perception of work-related stress. Additionally, past use of PN substances increased participants’ willingness to use them again [24]. Wiegel and colleagues showed that willingness was influenced by past experiences and that past use of PN substances was prevalent in 0.88 % of today’s university teachers; the low rates of willingness can be explained by the definition of the term “CE-drug” in their study to be “pharmaceuticals, especially prescription drugs”. Later in their paper, they define more precisely CE-drugs to be “prescription drugs to enhance work performance without any medical necessity” [24]. Furthermore, in our study, we found that only 11.8 % of the teachers knew about PN from their own time at university (which does not mean that they used it themselves). According to the low knowledge rate of PN from previous university experiences (11.8 %), and considering that own knowledge is a prerequisite for own use, one can assume that own use at university was very infrequent or even marginal, which is consistent with the results of Wiegel and colleagues. However, this past situation does not accurately represent the current situation, as several studies have shown high prevalence rates of PN of up to 20 % among today’s students [3–10]. One of the major problems explaining this range of 1 – 20 % in the prevalence rate is the heterogeneous definition of PN.

Furthermore, using an anonymizing technique, a survey study by Dietz and colleagues showed a past-year prevalence rate of 20 % among university students regarding the use of PN substances; this study defines PN substance use towards their participants as follows: “Substances for brain doping are pharmaceuticals or illegal drugs that you cannot buy in a drugstore and that were not prescribed to you to treat a disease. The only reason why you use this substance is to improve cognitive performance, such as attention, alertness, and mood. Examples are stimulant drugs (amphetamines), caffeine tablets, cocaine, methylphenidate, and mephedrone.” The most recent study by Liakoni and colleagues showed a prevalence rate of 13 % for “the use of prescription drugs, alcohol, or illegal drugs for CE” among secondary school students using a web-based design [9, 10].

The same study surveying 1400 university teachers as well as 3500 students revealed a significantly higher willingness to use PN substances among students than among teachers using a varying definition of a “hypothetical CE-drug” [24]. This underlies the situation mentioned previously and confirms the results presented here, that in the current student environment, the prevalence rates of PN substance use among students are significantly higher than in former times when today’s teachers were studying.

However, comparing the papers of Sattler and Wiegel to our sample is not directly possible because in our study, we investigated school teachers, while Sattler and Wiegel investigated university teachers (and students). Furthermore, definitions of PN are not directly comparable.

Wanja Wollf and colleagues surveyed a Jordanian sample of employees, of whom more than 700 were teachers, and the results of that study and those of the surveyed sample in our study (= teachers) are more or less directly comparable; however, Wolff et al. surveyed another country (Jordan), which was considered to represent an Arabic sample in their publication [25]. They provided the following definition of PN to the participants, that PN refers to “the use of drugs (like Ritalin or Modafinil) in order to improve cognitive capacity (e.g., alertness, concentration) without a medical indication to do so,” which was consistent with the definition used by other scientists to establish prevalence rates for students and employees [8, 9, 11, 18, 26–30]. However, the past-year prevalence rate of 26 % identified among teachers was not in line with the findings of the present study, in which only 11.8 % of the teachers knew about PN from their own experiences at university, and was not in line with the finding that teachers believed the prevalence of PN substance use to be very low. However, we did not ask teachers about their current use of PN substances in the study presented here.

An important prerequisite to using a certain substance for PN is the assumption that it is effective. Teachers in the 26 % past-year prevalence rate of Wollf et al. had to have assumed that PN substance use was effective; we found that 34.9 % of the teachers in our study were convinced that none of the so-called PN substance had pro-cognitive effects. This may be, at least in part, in accordance with the findings of Wolff and colleagues [25].

Most teachers in our study assumed that PN had a very low prevalence in schools. This seems to be dependent on the type of the substance, the surveyed group and the degree of anonymity during the survey and is at least to some extent a false assumption, as demonstrated by the double-digit lifetime and even past-year prevalence rates for OTC substances found in several past studies in Germany. In their systematic review, Wilens and colleagues demonstrated in 2008 a past-year prevalence rate of already 5–35 % regarding the general misuse of “stimulants prescribed for ADHD” among college students. Furthermore, Dietz and colleagues reported a 20 % past-year prevalence rate of the use of PN substances (PN definition above). Their prevalence rates were identified using the so-called RRT to guarantee anonymity to the participants [31–33]. Recent European survey studies (Switzerland) have confirmed rather high lifetime prevalence rates of PN substances use among students (Ott et al.: 6.2 %; Maier et al.: 13.8 %) [8, 34]. An even higher proportion was found in a recent survey of medical students in the US, namely a lifetime prevalence of stimulant use of 20 %, and a prevalence rate during the period of medical school of 15 % [6]. However, most of the previous and aforementioned studies investigated the use of PN substances among college or university students. We also asked teachers about their assumptions regarding school students/ pupils. An older survey study (2011) by Franke and colleagues surveyed high school students and demonstrated a lifetime prevalence of prescription drug use of 1.6 % and of illicit stimulants, 2.4 %; they also found significantly higher prevalence rates for caffeinated substances (lifetime prevalence rate of use of coffee: 53.2 %, of caffeinated drinks/ energy drinks: 39 % and of caffeine tablets: 10.5 %) [7, 35]. The low prevalence rate for high school students using prescribed and illicit drugs in the study of Franke and colleagues may be consistent with the teachers’ assumptions in the present study that PN substance use is not very widespread, as stated by the surveyed teachers. However, the way of dealing with PN in the present study to inform the participants is the use of any kind of substances by healthy people without medical need with the aim of increasing cognition. Regarding the underlying definition, the surveyed teachers show a false assumption.

Furthermore, the present survey study’s results revealed that teachers believed PN substance use to be significantly higher in college and university students than in school students/ pupils, which is in line with the aforementioned data.

Regarding the distribution of prevalence rates of the different types of substances, the assumptions of the surveyed teachers adhered to the “real” situation, showing significantly higher prevalence rates for caffeine and considerably lower prevalence rates for prescription and illicit drugs, which has been demonstrated in several previous studies [7, 35].

Regarding the effectiveness of PN substances, the scientific data are controversial. Nevertheless, roughly following the data on effectiveness, effectiveness seems to be limited to simple cognitive domains such as vigilance and attention, with (very) mild effects on higher cognitive domains such as memory; however, at least to the knowledge of the authors, there have been data demonstrating pro-cognitive effects on higher cognitive domains [27, 36–39]. However, a third of the surveyed teachers assume that there are no pro-cognitive effects and one-fourth thought that it would be possible to reach better grades (maybe staying awake all night long before an exam). This shows controversial assumptions and could mean uncertainty about the effects which is in line with the international study results mentioned before.

Regarding the risks of addiction, there have been lively and controversial debates in the scientific world as well as in the print and digital media [19, 40]. Michael Soyka and Andreas Franke addressed this important subject in a German review and concluded that caffeinated substances as well as Ginkgo biloba had (nearly) no risks of addiction, while the risk of addiction of misusing stimulants (AMPH, MPH) should not be underestimated [18]. This is consistent with the surveyed teachers’ evaluations of PN substances as having a high risk of addiction (only 1.2 % thought that none of the so-called PN substances could lead to addiction). Moreover, an older study showed that students using stimulants for PN were well aware of the risks of addiction [17, 21]. Additionally, stimulants that interact with dopamine have an important role in the association between the field of addiction and the field of PN, which emphasizes and confirms the assumptions and classifications of PN among the surveyed teachers [19, 41].

The vast majority of the surveyed teachers stated that “doping” in the world of sports should be forbidden; however, significantly fewer teachers would forbid “brain doping”. Compared to surveys among assisting personnel/ staff (e.g. coaches) in the world of sports, there are higher rates of this assisting personnel/ staff who voted in favor of anti-doping behavior. However, some of them admit that exceptions could be made under certain circumstances [42]. However, the more or less different relationship between teachers and their students on the one hand and coaches and athletes on the other hand should be considered [42, 43]. Beyond that, the success of both, teachers and coaches, can at least in part be measured by the success of the student/ athlete.

### Limitations

In addition to the aspects discussed above, some factors should be addressed that limit the explanatory power of this study. As with every survey study, the suitability of the content and length of the questionnaire and the likelihood of complete participation should be discussed. On the one hand, the questionnaire developed for this study was relatively long and thus led to plenty of information; on the other hand, the length led to a successively increasing drop-out rate of participation, reaching even one third for the last question which limits the explanatory and statistical power especially at the end of the questionnaire. Another important aspect of data interpretation is the degree of participants’ “honesty”: it should be mentioned, that surveys containing questions of stigmatizing behavior are answered much more honestly when techniques with a recognizable high degree of anonymity such as web-based studies are being used [44].

Furthermore, a web-based survey carries an inherent risk of participation bias because one cannot control for the type of participants or their knowledge, attitudes, and subjective aims when participating or not participating. Unfortunately, online surveys cannot control for this disproportion and for the introduction of response bias. Furthermore, participants could have informed others who were not invited to participate. Beyond that, like in many other web-based survey studies, data about a response rate cannot be estimated or even be given.

Nevertheless, to reach more than 250 participants throughout an entire federal state, an online survey was one of the best possible ways to collect “broad” data as a preliminary overview about an important topic associated with PN substance use being neglected so far. However, the generalizability has to be considered to be relatively low because of at least two reasons: Even though all of the schools in the federal state of Mecklenburg-Vorpommern were contacted, only 255 teachers participated, which is a relatively low number compared to the population of all teachers belonging to the contacted schools. Therefore, the survey only reflects the knowledge, assumptions and attitudes of a relatively small proportion of the teachers. Furthermore, the study has been conducted in only one federal state which is not representative for other federal states of Germany.

Another important aspect is the problem of the definition of “PN”; some researchers in this field use additional terms such as “soft enhancement” for the use of OTC substances or “brain doping” for the use of prescription and illicit drugs. For the participants of this study, we define PN to be the use of substances of any kind including OTC substances and energy drinks as well as prescription and illicit drugs by healthy people without medical need with the aim of increasing cognitive skills which is a relatively broad definition of PN.

## Conclusions

Teachers, as an integral part of the education of children and adolescents, often know about PN substances and mostly refuse their use being afraid about the risk of addiction. However, regarding effects as well as side effects of PN substances, teachers have very different opinions. Furthermore, they seem to underestimate the prevalence among their students and broach the topic infrequently. Teachers should be sensitized for high prevalence rates and should broach the topic of PN more frequently to their students in order to prevent potential misuse of PN substances.

## Abbreviations

ADHD: Attention deficit hyperactivity disorder
AMPH: Amphetamine
CE: Cognitive enhancement
CI: Confidence interval(s)
Df: Degrees of freedom
MDMA: (3,4-) Methylenedioxymethamphetamine (= ecstasy)
MPH: Methylphenidate
OR: Odds ratio(s)
OTC: Over the counter
PN: Pharmacological neuroenhancement
SD: Standard deviation(s)
